# Dental caries and associated factors: prevalence among children aged 6 to 12 years in 2023 in the city of Conakry, Guinea

**DOI:** 10.3389/froh.2026.1700504

**Published:** 2026-06-03

**Authors:** Aly Badara Nabe, Younoussa Sylla, Aly Badara Toure, Foromo Timothee Beavogui, Sidikiba Sidibe, Soriba Camara, Maladho Diaby, Castro Gbêmêmali Hounmenou, Jean Claude Guinan, Alexandre Delamou

**Affiliations:** 1 Department of Dentistry, Faculty of Health Sciences and Technology, Gamal Abdel Nasser University, Conakry, Guinea; 2 National Health Promotion Service, Ministry of Health and Public Hygiene, Conakry, Guinea; 3 African Center of Excellence for the Prevention and Control of Communicable Diseases (CEA-PCMT), Gamal Abdel Nasser University of Conakry, Guinea; 4 Department of Public Health, Faculty of Health Sciences and Technology, Gamal Abdel Nasser University, Conakry, Guinea; 5 Centre de Recherche et de Formation en Infectiologie de Guinée, Conakry, Guinea; 6 Département Informatique, Université de Labé, Labé, Guinea; 7 Department of Public Health, Faculty of Dentistry, Félix Houphouët Boigny University, Abidjan, Côte d'Ivoire; 8 National Center for Training and Research in Rural Health, Maferinyah, Forécariah, Guinea

**Keywords:** children aged 6–12, dental caries, gingivitis, hygiene, prevalence

## Abstract

**Objectives:**

To determine the prevalence of dental caries and analyze associated factors among children aged 6 to 12 years in Conakry, Guinea.

**Methods:**

This cross-sectional study was conducted on 869 children in Conakry, Guinea, from July to December 2023. Sociodemographic characteristics of children and their caregivers were evaluated. The DMFT index was used to measure caries prevalence. Explanatory variables included age, sex, education, district of residence, oral hygiene (OHI-S), gingival inflammation, orthodontic anomalies, and brushing habits. Descriptive, bivariate, and multivariable logistic regression analyses were performed to identify factors independently associated with caries prevalence.

**Results:**

Among 869 children, 72.8% had dental caries (DMFT > 0) and 27.2% were caries-free. The mean DMFT score was 2.24 ± 1.96. In multivariable analysis, four factors were independently associated with dental caries: age group 10–12 years (aOR=3.44; 95% CI: 2.41–4.90; *p* < 0.001), poor oral hygiene as assessed by OHI-S (aOR=1.54; 95% CI: 1.10–2.16; *p* = 0.013), gingival inflammation (aOR=1.85; 95% CI: 1.16–2.96; *p* = 0.010), and use of non-standard brushing materials (aOR=1.49; 95% CI: 1.00–2.23; *p* = 0.050).

**Conclusion:**

This study found a high prevalence of dental caries among children in urban Conakry, Guinea, driven by behavioral and clinical factors. Age group, poor oral hygiene, and gingival inflammation were identified as the main independent risk factors, with children aged 10–12 years being particularly vulnerable. These findings highlight the urgent need for targeted public health interventions, including school-based oral health education and improved access to affordable dental care to address this significant health burden.

## 1 Introduction

Dental caries, a multifactorial disease, constitute a growing global health burden (1). In 2019, approximately 3.58 billion people worldwide had caries on permanent teeth, while dental caries on primary teeth affected more than 530 million children (2). From 1990 to 2015, the total number of disability-adjusted life years lost due to dental caries increased significantly by 64% among school-age children. Dental caries not only cause physical pain but also impair children's overall health and quality of life, as well as that of their parents (3). Beyond being an indicator of well-being, a child's good oral health is crucial for their ability to interact with others and express their emotions (4). Children with dental caries are more likely to experience a loss of productivity, often reflected by poor academic performance. It is estimated that children miss more than 51 million school hours, equivalent to 10 million school days, due to caries and gingival diseases (5). Excluding indirect and intangible costs, dental caries also cost the global economy approximately 298 billion dollars in direct treatment, representing 4.6% of global health expenditures (6). In Africa, approximately half of the population suffers from dental caries, periodontal diseases, and tooth loss (7). African countries face a disproportionate burden of dental caries due to limited access to dental care, poor oral hygiene practices, inadequate oral health education, and a lack of preventive measures (8). Although data are scarce and the dental caries profile is not uniform, available evidence suggests an upward trend (2). In a systematic review of 22 articles on prevalence and 21 included articles from a DMFT meta-analysis, the overall prevalence of caries was 36% (29.4–41.7) and the overall mean DMFT was 1.09 (0.91–1.3) (9). In Tripoli, the overall prevalence was 74.7% (10). In sub-Saharan Africa, a 2014 study in Senegal revealed a mean prevalence of 51% among 12–15-year-olds (11). More recent data from Ghana in 2018 indicated a lower prevalence of 13.3% (2). In Nigeria (2023), the overall prevalence of dental caries was 23.0% (95% CI: 16–30) (12). Addressing a child's dental caries problem requires accessible and appropriate care, which is currently not a reality in many sub-Saharan African countries. These nations are characterized by a lack of oral health programs aimed at prevention and early access to care, which widens socioeconomic health disparities and unequal access to dental services.

In Guinea, epidemiological data on the dental and periodontal status of children are scarce. The few existing studies are partial and do not focus on specific groups of children. Although the etiological mechanisms of dental caries and their specifically behavioral determinants such as hygiene, diet, and early access to care are well understood, the availability of data on children's oral health remains a critical requirement for implementing appropriate public health strategies (13). It is in this context that this cross-sectional study was conducted, aimed at determining the prevalence of dental caries and analyzing associated factors among children aged 6 to 12 years in Conakry city.

## 2 Methods

### 2.1 Study area

This study was conducted in seventeen districts across three urban communes (Dixinn, Kaloum, and Matam) in Conakry, the capital city of Guinea. These communes were purposively selected due to their high concentration of low- to moderate-income households with children aged six to twelve years, in order to target populations considered particularly vulnerable to oral health problems. Conakry has a child population (aged zero to seventeen years) representing 43.8% of the total population and a multidimensional poverty index of 6.8% (14). The selected districts are characterized by limited access to oral health services, constrained availability of dental hygiene products, and suboptimal living conditions, all of which may increase the risk of dental caries

### 2.2 Study design and population

An analytical cross-sectional study was conducted between July and December 2023 among households in Conakry, Guinea. A two-stage sampling strategy was applied. In the first stage, the three communes of Dixinn, Kaloum, and Matam were purposively selected based on their sociodemographic characteristics and high proportion of households with children aged six to twelve years. In the second stage, eligible households (those with at least one child aged six to twelve years) were selected using systematic random sampling to reach a calculated sample size of 384 households. All eligible children within selected households were invited to participate. To limit intra-household correlation and preserve statistical independence, the number of enrolled children was capped at three per household; when more than three eligible children were present, random selection was performed. The final sample consisted of 869 children from 384 households.

### 2.3 Data collection

Data were collected using a standardized, structured questionnaire administered to the children's caregivers. The questionnaire included socio-demographic variables (child's age, sex, and school level, as well as selected household characteristics); behavioral variables related to oral hygiene practices and dietary habits; and clinical variables including caries experience, gingival inflammation, and orthodontic anomalies.

Several indicators related to household living conditions and caregiver characteristics (caregiver's education level, caregiver's occupation, household income, household size, asset ownership, and housing conditions) were collected as proxies of the socio-economic context; however, these indicators were not combined into a composite socio-economic index.

### 2.4 Oral clinical examination

The oral health status of each included child was assessed using standardized examination materials including an oral mirror, exploratory probes No. 6 and No. 17, a WHO periodontal probe, and tweezers, supplemented by artificial lighting.

Caries experience was measured using the DMFT index (Decayed, Missing, and Filled Teeth), recorded according to the diagnostic criteria of the World Health Organization (15). Results were expressed in two ways: caries prevalence, defined as the proportion of children presenting at least one affected tooth (DMFT > 0) versus unaffected (DMFT=0), and the mean DMFT score calculated for the entire sample.

Oral hygiene was quantified according to the Simplified Oral Hygiene Index (OHI-S) of Greene and Vermillion, combining a dental plaque index and a calculus index (16). Six dental surfaces were evaluated (buccal surfaces of teeth 16, 11, 26, 31 and lingual surfaces of teeth 36, 46). The overall OHI-S score corresponded to the sum of the plaque and calculus indices divided by the number of surfaces examined. Oral hygiene was categorized as follows: good (OHI-S: 0.0–1.2), fair (OHI-S: 1.3–3.0), or poor (OHI-S: 3.1–6.0). In accordance with WHO recommendations, in case of absence or incomplete eruption of a selected tooth, the adjacent ipsilateral tooth was substituted.

Gingival inflammation was assessed using the Gingival Index of Silness and Löe (16). Six reference teeth were examined (16, 12, 24, 36, 32, 44), with a score from 0 to 3 assigned to each of the four dental surfaces (mesial, distal, buccal, and lingual/palatal). The mean gingival score was calculated and interpreted according to the following categories: absence of inflammation (0), mild inflammation (0.1–1.0), moderate inflammation (1.1–2.0), and severe inflammation (2.1–3.0).

Orthodontic anomalies were defined as any deviation from intra-arch dental alignment or inter-arch occlusal relationships compared to normal physiological occlusion.

### 2.5 Examiner calibration

Before the start of data collection, a calibration training session was organized to ensure concordance and reproducibility of clinical assessments. Intra-examiner and inter-examiner reliability was assessed by calculating Cohen's Kappa coefficient, with an acceptability threshold set at κ ≥ 0.75, indicating significant agreement.

### 2.6 Statistical analysis

Statistical analyses were performed using R software (version 4.5.2). Categorical variables were summarized as frequencies and percentages, while continuous variables were expressed as means and standard deviations. Bivariate associations were assessed using Pearson's chi-square test, with a significance threshold set at *p* < 0.05.

Multivariate binary logistic regression was used to identify factors independently associated with dental caries prevalence. Variables included demographic characteristics, oral hygiene indicators, clinical parameters, and behavioral factors. Model selection was performed using a backward stepwise procedure based on the Quasi-likelihood Information Criterion (QIC), as required by GEE models. Adjusted odds ratios (aORs) with 95% confidence intervals were reported. Model performance was assessed using the Hosmer-Lemeshow goodness-of-fit test, variance inflation factors (VIF < 5) to examine multicollinearity, and Receiver Operating Characteristic (ROC) curve analysis to estimate discriminant ability. A Classification and Regression Tree (CART) analysis was performed as a complementary exploratory approach. The final GEE model (QIC=941.1) identified four factors independently associated with dental caries prevalence: the 10–12 years age group, poor oral hygiene (OHI-S > 1.2 (fair or poor hygiene), presence of gingival inflammation, and use of non-standard brushing materials. The model showed adequate fit (Hosmer–Lemeshow test: *p* = 0.966) and acceptable discriminant ability [AUC=0.69 (95% CI: 0.65–0.72)] according to Hosmer and Lemeshow criteria (0.60–0.70 = acceptable; 0.70–0.80 = good; > 0.80 = excellent). No multicollinearity was detected among the retained variables (VIF < 1.10 for all variables). Data completeness was assessed prior to analysis. No missing data were observed for the variables of interest, allowing the inclusion of all 869 children in the bivariate and multivariable analyses.

### 2.7 Ethical statement

The study protocol was approved by the National Ethics Committee for Health Research (CNERS) of Guinea (No. 072/CNERS/23). Free and informed consent was obtained from patients before data collection, and all information was collected anonymously and confidentially.

## 3 Results

This study targeted children aged 6–12 years, surveyed in 384 households in Conakry city. The analyzed sample thus comprised 869 children, distributed among the communes of Dixinn (*n* = 219), Kaloum (*n* = 407) and Matam (*n* = 243).

### 3.1 Socio-demographic characteristics

The study population comprised 869 children from the communes of Dixinn, Kaloum, and Matam. Participants were aged 6 to 12 years and enrolled between the 1st and 7th school year, with 92.8% at the primary level. The sex ratio was 1.1 in favor of boys. The mean age of children was 8.96 ± 2.06 years (Table 1).

**Table 1:** Sociodemographic and household characteristics of children and families.

Characteristics	*N*	%
Child characteristics	***N*** **=** **869 children**	
Age (years)		
Mean ± SD	**8.96** **±** **2.06**	
6–9 years	516	59.4
10–12 years	353	40.6
Sex		
Female	411	47.3
Male	458	52.7
Sex ratio (M/F)	**1.1**	
District of residence		
Dixinn	219	25.2
Kaloum	407	46.8
Matam	243	28.0
Child's education level		
Primary school	806	92.8
Middle school	63	7.2
Household characteristics	***N*** **=** **384 households**	
Caregiver's level of education		
Out of school	145	37.8
School-going	239	62.2
Household size		
Mean ± SD	**7.2** **±** **3.1**	
Median (IQR)	**7 (5–9)**	
Small (≤ 4 persons)	82	21.4
Medium (5–7 persons)	156	40.6
Large (≥ 8 persons)	146	38.0
Household crowding		
Persons per room, mean ± SD	**2.8** **±** **1.4**	
Not crowded (< 2 persons/room)	124	32.3
Crowded (≥ 2 persons/room)	260	67.7
Housing conditions		
Adequate	142	37.0
Inadequate	242	63.0

SD, standard deviation; IQR, interquartile range.

Bold values indicate statisticallysignificant results (*p* < 0.05).

### 3.2 Hygiene habits

Among the 869 children, the mean OHI-S score was 1.82 ± 0.84. Good oral hygiene (score ≤ 1.2) was observed in only 37.1% of participants (*n* = 322), while the majority, 62.9% (*n* = 547) presented with fair or poor hygiene (score > 1.2), indicating a predominantly insufficient oral hygiene status in this population. A toothbrush was the most commonly used brushing tool (79.4%, *n* = 690), followed by a traditional chewing stick (Gbèssè, 12.1%, *n* = 105), finger with charcoal (8.3%, *n* = 72), and no brushing (0.2%, *n* = 2). Most children reported brushing once daily (69.7%), whereas 27.4% brushed twice daily and 2.9% brushed three times daily. Brushing was predominantly performed before meals (92.1%), with 7.9% brushing after meals (Table 2).

**Table 2:** Distribution of children according to the degree of oral hygiene (*N* = 869).

Characteristics	*N* = 869	%
OHI-S		
Mean ± SD	**1.82** **±** **0.84**	
Good hygiene (≤1.2)	322	37.1%
Fair/Poor hygiene (>1.2)	547	62.9%
Brushing materials		
Toothbrush	690	79.4
Gbèssè (traditional chewing stick)	105	12.1
Finger + charcoal	72	8.3
Does not brush	2	0.2
Brushing frequency		
Once daily	606	69.7
Twice daily	238	27.4
Three times daily	25	2.9
Timing of brushing		
Before meals	800	92.1
After meals	69	7.9

OHI-S, oral hygiene index simplified; SD, standard deviation.

Bold values indicate statistically significant results (*p* < 0.05).

### 3.3 Caries lesions

Among 869 children, 72.8% had dental caries (DMFT > 0), while 27.2% were caries-free. Based on DMFT categories, 47.1% had mild to moderate caries (DMFT 1–3) and 25.8% had severe caries (DMFT 4–9); the mean DMFT score was 2.24 ± 1.96. The presumed onset of caries was reported as >1 year in 16.8%, 7–12 months in 11.6%, and 1–6 months in 14.2% of children; onset was unknown in 28.7%, and 28.8% had no carious teeth. Gingival inflammation was present in 20.3% of participants, and orthodontic anomalies were observed in 17.5% (Table 3).

**Table 3:** Distribution of children aged 6–12 years according to caries prevalence and severity.

Variables	*N* = 869	%
Presence of dental caries		
Yes (DMFT > 0)	633	72.8
No (DMFT=0)	236	27.2
Presumed onset of caries		
> 1 year ago	146	16.8
7 months to 1 year	101	11.6
1 to 6 months	123	14.2
No carious teeth	250	28.8
Unknown	249	28.7
DMFT index categories		
0 (no caries)	236	27.2
1–3 (mild to moderate)	409	47.1
4–9 (severe)	224	25.8
Mean DMFT ± SD	**2.24** **±** **1.96**	
Gingival inflammation		
Absent	693	79.7
Present	176	20.3
Orthodontic anomalies		
Absent	717	82.5
Present	152	17.5

DMFT, decayed, missing, filled teeth; SD, standard deviation.

Bold values indicate statistically significant results (*p* < 0.05).

In bivariate analysis, age, oral hygiene status, gingival inflammation, and several household socioeconomic factors were significantly associated with dental caries prevalence. Children aged 10–12 years had a significantly higher likelihood of dental caries compared with those aged 6–9 years (aOR=3.42; 95% CI: 2.41–4.84; *p* < 0.001). Children attending primary school had lower odds of dental caries compared with those in middle school, though this association did not reach statistical significance (OR = 0.61; 95% CI: 0.32–1.17; *p* = 0.133). Children who used non-standard brushing materials or did not brush their teeth had significantly higher odds of dental caries compared with those using a toothbrush (OR = 1.49; 95% CI: 1.01–2.22; *p* = 0.045). Children with fair or poor oral hygiene (OHI-S > 1.2) had significantly higher odds of dental caries compared with those with good or excellent oral hygiene (OR = 1.82; 95% CI: 1.33–2.48; *p* < 0.001). The presence of gingival inflammation was significantly associated with higher odds of dental caries (OR = 2.19; 95% CI: 1.40–3.44; *p* < 0.001), whereas orthodontic anomalies were not. At the household level, children whose caregivers had no formal education were more likely to have dental caries (OR = 1.03; 95% CI: 0.78–1.38; *p* = 0.818). Living in a large household (> 10 persons) was associated with reduced odds of caries (OR = 0.56; 95% CI: 0.33–0.96; *p* = 0.036) (Table 4).

**Table 4:** Bivariate analysis of factors associated with dental caries prevalence among children aged 6–12 years.

Characteristics	No caries *N* = 236 (27.2%)	Caries present *N* = 633 (72.8%)	OR [95% CI]	*p*-value
Child characteristics (*N* = 869 children)				
Age group				**< 0.001**
6–9 years	186 (36.0%)	330 (64.0%)	Ref.	
10–12 years	50 (14.2%)	303 (85.8%)	3.46 [2.42–4.94]	
Sex				0.660
Female	115 (28.0%)	296 (72.0%)	Ref.	
Male	121 (26.4%)	337 (73.6%)	1.08 [0.80–1.47]	
District of residence				0.121
Dixinn	65 (29.7%)	154 (70.3%)	Ref.	
Kaloum	117 (28.7%)	290 (71.3%)	1.05 [0.73–1.50]	
Matam	54 (22.2%)	189 (77.8%)	1.50 [1.02–2.20]	
Child's education level				0.175
Primary school	224 (27.8%)	582 (72.2%)	Ref.	
Middle school	12 (19.0%)	51 (81.0%)	1.65 [0.89–3.07]	
Behavioral factors				
Brushing materials				**0**.**057**
Toothbrush	198 (28.7%)	492 (71.3%)	Ref.	
Other/does not brush	38 (21.2%)	141 (78.8%)	1.49 [1.02–2.18]	
Brushing frequency				0.515
Once daily	169 (27.9%)	437 (72.1%)	Ref.	
Twice or more daily	67 (25.5%)	196 (74.5%)	1.13 [0.81–1.57]	
Clinical factors				
OHI-S (oral hygiene)				**< 0.001**
Good/Excellent (≤1.2) *n* = 322	112 (34.8%)	210 (65.2%)	Ref.	
Fair/Poor (>1.2) *n* = 547	124 (22.7%)	423 (77.3%)	1.82 [1.33–2.48]	
Orthodontic anomalies				0.876
Absent	196 (27.3%)	521 (72.7%)	Ref.	
Present	40 (26.3%)	112 (73.7%)	1.05 [0.72–1.54]	
Gingival inflammation				**< 0.001**
Absent	207 (29.9%)	486 (70.1%)	Ref.	
Present	29 (16.5%)	147 (83.5%)	2.19 [1.40–3.44]	
Household socioeconomic factors (*N* = 384 households)				
Caregiver's education				0.904
Has formal education	133 (27.4%)	352 (72.6%)	Ref.	
No formal education	103 (26.8%)	281 (73.2%)	1.03 [0.78–1.38]	
Household size				0.125
Small ≤ 5 persons	92 (25.2%)	273 (74.8%)	Ref.	
Medium 6–10 persons	120 (27.3%)	320 (72.7%)	0.90 [0.66–1.22]	
Large > 10 persons	24 (37.5%)	40 (62.5%)	0.56 [0.33–0.96]	

OR, crude odds ratio estimated by GEE (generalized estimating equations); 95% CI, 95% confidence interval; p, Wald test; OHI-S, simplified oral hygiene index. Bold: *p* < 0.05.

Bold values indicate statistically significant results (*p* < 0.05).

In the GEE multivariable model, four child-level factors were independently associated with the presence of dental caries. Children aged 10–12 years had significantly higher odds of dental caries compared with those aged 6–9 years (aOR=3.44; 95% CI: 2.41–4.90; *p* < 0.001). Fair or poor oral hygiene, as assessed by the OHI-S, was also independently associated with dental caries (aOR=1.54; 95% CI: 1.10–2.16; *p* = 0.013). Similarly, children presenting with gingival inflammation had nearly twice the odds of dental caries compared with those without inflammation (aOR=1.85; 95% CI: 1.16–2.96; *p* = 0.010). Additionally, the use of non-standard brushing materials was retained in the final model, showing a borderline association with dental caries (aOR=1.49; 95% CI: 1.00–2.23; *p* = 0.050). Sex, district of residence, brushing frequency, orthodontic anomalies, and education level were not retained after backward selection (Table 5). The overall mean DMFT score among the 869 children was 2.24 ± 1.96. Age was strongly associated with caries severity, with children aged 10–12 years presenting a significantly higher mean DMFT than those aged 6–9 years (2.91 ± 1.98 vs. 1.78 ± 1.81), corresponding to a mean difference of +1.13 (95% CI: 0.87–1.39; *p* < 0.001). No significant difference in mean DMFT was observed by sex (female: 2.13 ± 1.90; male: 2.34 ± 2.01; mean difference: +0.21, 95% CI: −0.05 to 0.47; *p* = 0.107). District of residence was significantly associated with mean DMFT (*p* = 0.013), with children from Kaloum and Matam presenting higher scores than those from Dixinn [mean differences: +0.43 (95% CI: 0.13–0.74; *p* = 0.005) and +0.47 (95% CI: 0.15–0.79; *p* = 0.004), respectively]. Children with fair or poor oral hygiene had a substantially higher mean DMFT compared with those with good or excellent hygiene (2.50 ± 2.04 vs. 1.80 ± 1.73), yielding a mean difference of +0.69 (95% CI: 0.44–0.96; *p* < 0.001). Similarly, the presence of gingival inflammation was associated with greater caries severity (2.75 ± 1.91 vs. 2.11 ± 1.95), with a mean difference of +0.64 (95% CI: 0.32–0.96; *p* < 0.001). At the household level (*N* = 384 households), none of the socioeconomic variables reached statistical significance for mean DMFT differences. Children from high-income households presented a mean difference of −0.03 (95% CI: −0.49 to 0.42; *p* = 0.895) compared with those from low-income households, a difference that was not statistically significant (Table 6).

**Table 5:** Multivariable logistic regression using generalized estimating equations (GEE): factors associated with dental caries among children.

Characteristics	Crude OR [95% CI]	Adjusted OR [95% CI]	*p*-value	Sig.
Age group			< 0.001	
6–9 years	Ref.	Ref.		
10–12 years	3.46 [2.42–4.94]	3.44 [2.41–4.90]	< 0.001	*
Brushing materials			0.050	
Toothbrush	Ref.	Ref.	.	
Other/does not brush	1.49 [1.02–2.18]	1.49 [1.00–2.23]	0.050	*
OHI-S (oral hygiene)			0.013	
Good/Excellent (≤1.2) *n* = 322	Ref.	Ref.	.	
Fair/Poor (>1.2) *n* = 547	1.82 [1.33–2.48]	1.54 [1.10–2.16]	0.013	*
Gingival inflammation			0.010	
Absent	Ref.	Ref.	.	
Present	2.19 [1.40–3.44]	1.85 [1.16–2.96]	0.010	*

^a^OR, adjusted odds ratio estimated by GEE; 95% CI, 95% confidence interval; OHI-S, simplified oral hygiene index

*p < 0.05.

**Table 6:** Distribution of mean DMFT index according to sociodemographic and clinical characteristics of children aged 6 to 12 years.

Variable	*n* (%)	Mean DMFT ± SD	Mean difference [95% CI]	*p*
Overall	**869** (**100%)**	**2.24** ± **1.96**	**—**	**—**
Child characteristics (*N* = 869)				
Age group				
6–9 years	516 (59.4%)	1.78 ± 1.81	Ref.	
10–12 years	353 (40.6%)	2.91 ± 1.98	+1.13 [0.87–1.39]	**< 0.001**
Sex				
Female	411 (47.3%)	2.13 ± 1.90	Ref.	
Male	458 (52.7%)	2.34 ± 2.01	+0.21 [−0.05–0.47]	0.108
District of residence				
Dixinn	219 (25.2%)	1.90 ± 1.65	Ref.	
Kaloum	407 (46.8%)	2.34 ± 2.14	+0.43 [0.13–0.74]	**0**.**009**
Matam	243 (28.0%)	2.37 ± 1.88	+0.47 [0.15–0.79]	**0**.**005**
School level				
Primary school	806 (92.8%)	2.18 ± 1.94	Ref.	
Middle/High school	63 (7.2%)	2.97 ± 2.02	+0.79 [0.27–1.30]	**0**.**002**
Behavioral factors				
Brushing material				
Toothbrush	690 (79.4%)	2.17 ± 1.97	Ref.	
Other/no brushing	179 (20.6%)	2.52 ± 1.90	+0.35 [0.04–0.67]	**0**.**032**
Clinical factors				
Oral Hygiene Status				
Good/Excellent (≤1.2) *n* = 322	322 (37.1%)	1.80 ± 1.73	Ref.	
Fair/Poor (>1.2) *n* = 547	547 (62.9%)	2.50 ± 2.04	+0.69 [0.44–0.95]	**< 0.001**
Gingival inflammation				
Absent	693 (79.7%)	2.11 ± 1.95	Ref.	
Present	176 (20.3%)	2.75 ± 1.91	+0.64 [0.32–0.96]	**< 0.001**
Household socioeconomic factors				
Household income				
Low	508 (58.5%)	2.20 ± 1.94	Ref.	
Moderate	289 (33.3%)	2.33 ± 2.02	+0.13 [−0.16–0.42]	0.371
High	72 (8.3%)	2.17 ± 1.84	−0.03 [−0.49–0.42]	0.895

DMFT, decayed, missing, filled teeth; SD, standard deviation; 95% CI, 95% confidence interval. Bold: *p* < 0.05.

**Figure 1:**
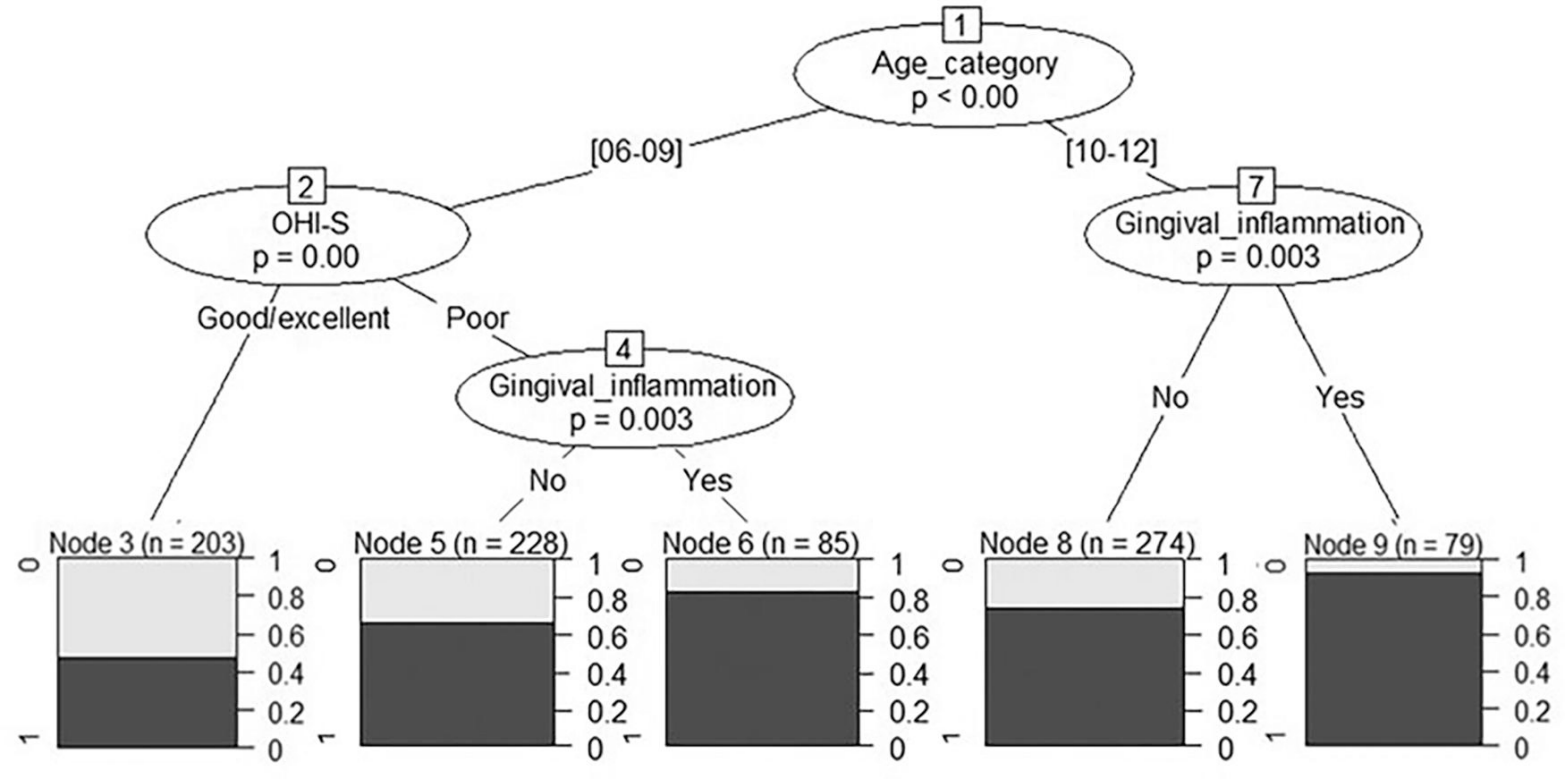
Correlation analysis through the decision tree, dental caries prevalence among children aged 6–12 years Conakry 2023 (*N* = 869).

The decision tree confirms that age, OHI-S, and gingival inflammation are the main factors associated with dental caries prevalence. Age category was the primary splitting variable (Node 1; *p* < .001), distinguishing children aged 6–9 years (*n* = 516) from those aged 10–12 years (*n* = 353). Among children aged 6–9 years, OHI-S was the second splitting variable (Node 2; *p* = .006), with 322 children (37.1%) presenting good or excellent hygiene and 547 (62.9%) presenting fair or poor hygiene. Older children (10–12 years), those with poor oral hygiene, and those with gingival inflammation had a higher probability of dental caries than younger children (6–9 years). Within the 10–12 years age group, children with gingival inflammation had the highest probability of dental caries (Node 9, *n* = 79, 92.4%; *p* = 0.057) compared with those without gingival inflammation (Node 8, *n* = 274, 83.9%). Among 6–9-year-olds with poor OHI-S, the presence of gingival inflammation further increased the probability of dental caries (Node 6, *n* = 85, 77.6%; *p* = 0.035) relative to those without gingival inflammation (Node 5, *n* = 228, 64.9%). Children aged 6–9 years with good or excellent OHI-S had the lowest probability of dental caries across all terminal nodes (Node 3, *n* = 203, 57.1%) (Figure 1).

## 4 Discussion

The prevalence of dental caries in children represents a significant and growing health burden, particularly in low- and middle-income countries. Since almost all risk factors for dental caries are modifiable, their development can be prevented by appropriate public health interventions. As with many non-communicable diseases, socioeconomic, behavioral, and environmental factors play a major role in prevention. Our study population consisted of 869 children from three communes of Conakry (Table 1), aged 6 to 12 years.

Although 79.4% of the children used a toothbrush, the prevalence of dental caries was 72.8%. Despite the reported use of a toothbrush by 79.4% of children, the prevalence of caries reached 72.8%, suggesting that possession of equipment alone was not sufficient to prevent the disease. This divergence could be explained by inadequate brushing technique, insufficient frequency (only 27.4% brush ≥2 times/day), and limited access to fluoridated toothpaste in Conakry's socioeconomic context. These results demonstrated the importance of interventions targeting not only access to materials, but also the quality and frequency of brushing, as well as the use of fluoridated toothpaste. A similar dissociation between toothbrush use and preventive effectiveness was observed in 2023 in Singapore (17), where good oral hygiene practices were linked to lower caries rates. A majority of children (92.1%) brushed their teeth before meals, and only 8% after. This pre-meal brushing habit is similar to results reported in other studies in Sudan (18) and Nigeria (4). However, this practice is not ideal for removing food particles and plaque accumulated during the day, which may explain the high caries rates despite reported high brushing frequency. Differences in results could be due to varying levels of knowledge, attitudes, and practices regarding oral hygiene, particularly as our study was conducted among children from very disadvantaged households. Additionally, differences in sample size and high prevalence of dental caries could also explain the disparity.

The overall prevalence of dental caries was 72.8%, with a mean DMFT score of 2.24. This high prevalence may be attributed to children's and parents’ oral hygiene behaviors, their living environment, the lack of community-based caries prevention policies, and the high cost and limited availability of dental care services. The urban setting of the study may also explain changes in dietary habits, particularly the increased consumption of sugary foods. Children aged 10–12 years exhibited a caries prevalence three times higher than those aged 6–9 years. This finding is consistent with studies reporting a high burden of dental caries among older children, notably in Uganda (19) in East Africa and in Indonesia (20) in Southeast Asia. Conversely, the prevalence observed in our study was higher than that reported in Khartoum, Sudan (18), Accra, Ghana (2), as well as in studies conducted in Ethiopia (21) and another study from Tanzania (22). However, our findings remained lower than those reported in Saudi Arabia, where a high prevalence of dental caries was observed among both younger and older children (23). These disparities across studies may be explained by differences in toothbrushing techniques and frequency, socioeconomic status, household living standards, and the organization of local health systems, particularly the availability and accessibility of preventive and curative oral health services.

An increased susceptibility to dental caries was observed. In bivariate analysis, this susceptibility was statistically significant for age, OHI-S, gingival inflammation, and brushing materials. However, multivariate analysis demonstrated a strong and independent association only with age, OHI-S, and gingival inflammation, while brushing materials showed a borderline association. These findings are consistent with studies conducted in Singapore (17), Italy (24) and Tel Aviv, Israel, where dental plaque and gingivitis were directly associated with an increased risk of dental caries (25). In China, a significant relationship between the plaque index and dental caries has been demonstrated (26), while studies conducted in India (27) and Indonesia reported that children with high dental plaque indices had a 3.3-fold higher risk of severe dental caries compared with those presenting low plaque indices (7).

Stratified analysis showed that the mean DMFT index in this study reflected a substantial severity of dental caries among children aged 6–12 years in Conakry, corresponding to approximately two decayed teeth per child on average. These findings were higher than those reported in Ghana (2) but lower than the mean DMFT observed in India (25). A significant difference according to age group was observed, with children aged 10–12 years presenting a mean DMFT approximately twice as high as those aged 6–9 years (*p* < 0.001). This age-related increase may be explained by cumulative exposure to risk factors, progressive autonomy in oral hygiene practices, and the transition period toward permanent dentition, as previously reported in the literature (28).

Children with good oral hygiene exhibited a significantly lower mean DMFT compared with those presenting poor oral hygiene (*p* < 0.001). This finding may be attributed to the lack of parental supervision during toothbrushing and inadequate oral hygiene practices. These results are consistent with observations reported by Utami in Indonesia (20), where the absence of parental supervision during toothbrushing was similarly identified as a significant risk factor for dental caries. Gingival inflammation was also significantly associated with higher mean DMFT scores, with children presenting gingivitis showing a substantially higher mean DMFT than those without gingival inflammation (*p* < 0.001). These findings are in agreement with the results reported by Anusha D. et al. (27).

The decision tree confirms the association between caries prevalence and the factors of age, OHI-S, and gingival inflammation. It highlights that caries prevalence increases with age, and that poorer oral hygiene exposes children to higher caries risk. Among children aged 10–12 years, the presence of gingival inflammation was associated with the highest caries prevalence observed across all terminal nodes, compared with a slightly lower but still elevated prevalence among those without inflammation. Among children aged 6–9 years with poor OHI-S, gingival inflammation further increased caries prevalence relative to those without inflammation, while children of the same age group with good or excellent OHI-S showed the lowest caries prevalence across all nodes. The increased vulnerability to dental caries in this age group may be because it coincides with the transition from primary to permanent dentition. This transition is characterized by the eruption of less mineralized and immature teeth, which are more vulnerable to caries. The difficulty children encounter in effectively cleaning their teeth during this mixed dentition period increases plaque retention and caries development. This period of life also corresponds to a phase when older children develop personal values and autonomy, which can lead to reduced parental supervision of oral hygiene and all factors associated with caries. This hypothesis is corroborated by a study conducted in Nigeria (4) and by other research suggesting that parental involvement in oral hygiene practices positively influences the maintenance of oral health (29). The high prevalence of caries in our study group, particularly among older children, suggests an urgent need for targeted public health interventions that address not only individual hygiene practices but also the broader social and economic determinants of health in the Conakry population.

This study has several limitations that should be considered when interpreting its findings. First, the cross-sectional design prevents the establishment of causal relationships between the identified risk factors and dental caries. The temporal sequence between exposure and outcome cannot be determined, and reverse causality cannot be excluded. Second, the sample was drawn exclusively from three urban communes of Conakry, which limits the generalizability of findings to rural areas and to Guinea as a whole, where oral health infrastructure, dietary patterns, and hygiene practices may differ substantially. Third, caries diagnosis relied solely on clinical examination without radiographic assessment. Given that interproximal and early lesions are not detectable clinically, this approach likely underestimates the true burden of dental caries in this population. Fourth, data on brushing habits, dietary practices, and fluoride exposure were collected through caregiver-reported questionnaires, introducing potential social desirability bias, particularly regarding oral hygiene frequency and technique. Fifth, several household-level socioeconomic variables — including housing conditions and household crowding — could not be included in the final GEE model due to inability to reproduce their original definitions from the available dataset; this may have led to residual confounding by unmeasured socioeconomic factors. Finally, the study was conducted in purposively selected communes characterized by low-to-moderate income households, which may have introduced selection bias and further limits the representativeness of the findings.

Notwithstanding these limitations, this study presents notable strengths. It is the first large-scale analytical study on dental caries among children aged 6–12 years in Conakry, based on a representative household sample of 869 children from 384 households. Clinical assessments used standardized WHO indices (DMFT, OHI-S, Gingival Index of Silness and Löe), and examiner calibration ensured reliability of measurements. The use of Generalized Estimating Equations (GEE) appropriately accounted for the non-independence of children within the same household, and the complementary CART analysis provided an accessible visual representation of the interaction between risk factors.

## 5 Conclusion

This study highlights a particularly high prevalence of dental caries, affecting 72.8% of children aged 6 to 12 years in Conakry. Age, inadequate oral hygiene, gingival inflammation, and the use of non-standard brushing materials emerged as the main associated factors. Children aged 10 to 12 years showed a markedly higher vulnerability. These findings underscore the need for targeted interventions to reduce the burden of dental caries in this population.

It is important to strengthen school-based oral hygiene education programs, implement regular supervised toothbrushing sessions using appropriate materials, and improve access to preventive care through community outreach initiatives. Encouraging greater parental involvement in monitoring toothbrushing practices and reducing sugar intake is also essential. At the national level, prioritizing the integration of oral health into public health policies and improving access to equitable and affordable dental services would help reduce the burden of dental caries among children.

## Data Availability

The raw data supporting the conclusions of this article will be made available by the authors, without undue reservation.
